# Degree and Complexity of Non-conscious Emotional Information Processing – A Review of Masked Priming Studies

**DOI:** 10.3389/fnhum.2021.689369

**Published:** 2021-06-22

**Authors:** Michaela Rohr, Dirk Wentura

**Affiliations:** Department of Cognitive Psychology, Saarland University, Saarbrücken, Germany

**Keywords:** (non-)consciousness, emotion, affect, masked priming, evaluative priming

## Abstract

Whether and to what degree information can be processed non-consciously has been a matter of debate since the emergence of psychology as a science. Emotional information, in particular, has often been assumed to have a privileged status because of its relevance for well-being and survival (e.g., to detect a threat). Indeed, many studies have explored non-conscious processing of evaluative (i.e., “emotional” in a broad sense) or emotional (e.g., facial expressions) features using the “silver bullet” of non-consciousness research – the masked sequential priming paradigm. In its prototypical form, this paradigm involves the categorization of target stimuli according to valence (e.g., is the target positive or negative?). Each target is preceded by a briefly presented prime that is followed by a mask to constrain awareness. Non-conscious processing is inferred from subtle influences of the prime on target processing, that is, whether responses are faster if prime and target are valence-congruent or not. We will review this research with a focus on three questions: first, which methods are used in this area to establish non-conscious processing? Second, is there evidence for non-conscious extraction of evaluative information? Third, is there evidence for non-conscious processing beyond a simple valence (positive/negative) discrimination, for example, processing of emotion-specific information? We will highlight important current debates and potential directions in which the field will move in the future.

## Introduction

Non-conscious processing of emotional information has been a focus of psychological research since its inception. With the emergence of computerized technology and the availability of new methods, non-conscious processing has become an important empirical issue in both consciousness research and emotion psychology. As in other fields, the conclusions drawn from the empirical data depend on the methods used. In the field of non-conscious emotion processing, the most prominent method is certainly the masked presentation of evaluative, affective, or emotion-related stimuli and the behavioral assessment of their influences. In the most prototypical paradigm, the evaluative priming paradigm, participants perform a categorization task (i.e., valence categorization: is the target positive or negative?) with clearly visible, task-relevant target stimuli (i.e., the target is shown for 500 ms or longer, often until response). Of critical importance, each task-relevant target stimulus is preceded by an evaluative prime stimulus that is presented very briefly (i.e., typical durations range from 20 to 50 ms) and then immediately followed by a masking stimulus (see Fazio et al., 1986; Greenwald et al., 1996, for the introduction of the paradigm in its unmasked form). In the ideal case, the prime remains outside of conscious awareness due to this presentation mode. Non-conscious processing is then inferred from the presence of an influence of the prime on target processing, that is, whether responses or error rates (or other dependent variables such as event-related potentials in brain-electrical activity or psychophysiological variables) differ depending on the relation or match/non-match between prime and target. Faster processing and/or fewer errors in congruent compared to incongruent prime-target trials (i.e., trials in which the valence of prime and target match versus mismatch) then indicate non-conscious processing of the prime.

The present review provides a comprehensive overview of the state of the art of research using this paradigm, as well as open questions and current controversies. We will focus on three questions: First, which assessment methods are used in this area to establish non-conscious processing? Second, is there evidence for non-conscious extraction of evaluative information? Third, is there evidence for non-conscious processing beyond a simple positive/negative discrimination? For example, is there evidence for non-conscious processing of emotion-specific information?

Please note that several meta-analyses and reviews have appeared in recent years in closely related fields and with different foci; these have covered the non-conscious processing of semantic information (Van den Bussche et al., 2009), the automatic but conscious processing of evaluative information (Herring et al., 2013), the neuronal basis of non-conscious emotion processing (Tamietto and DeGelder, 2010), physiological influences of non-consciously processed negative affective stimuli (van der Ploeg et al., 2017), and the non-conscious processing of threat stimuli (Hedger et al., 2016). Here, we will not reassess the evidence already reviewed in those articles; however, we will refer to these works where relevant for our overview. However, interestingly, no meta-analysis or review on masked evaluative or emotion processing exists.

Additionally, note that masked evaluative priming research lies at the intersection of research on fast, non-intentional processing of evaluative/affective features and research on masked priming in general (Marcel, 1983; Vorberg et al., 2003; Van den Bussche et al., 2009; Ansorge et al., 2014). For colleagues working in the latter field, the use of evaluative/valenced primes and targets is only one of many possible task instantiations using polarized semantic categories. Other possibilities include use of gender (e.g., categorization of male vs. female first names; e.g., Draine and Greenwald, 1998), size (e.g., categorization of small vs. large objects or animals; e.g., Pohl et al., 2010), or animacy (e.g., categorization of living vs. non-living things; e.g., Klinger et al., 2000). Against this backdrop, singling out the work on evaluative priming might appear somewhat arbitrary; however, we will discuss later whether or not the non-conscious processing of evaluative/emotional information should be considered “special” in some ways (see section “Is It ‘Cold’ Cognitive Processing or Are ‘Hot’ Emotion-Related Processes Involved?”).

## Non-Conscious Processing of Affect in Priming Research

**FIGURE 1:**
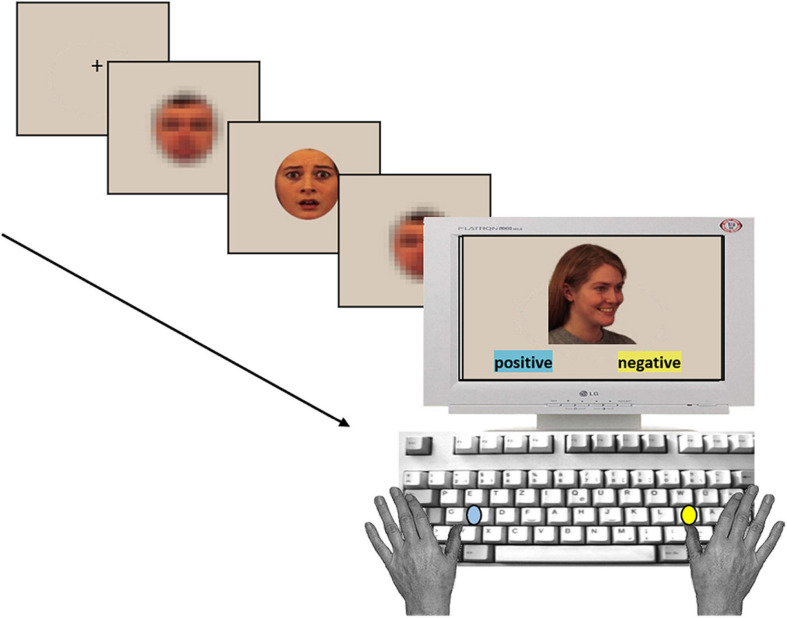
Trial sequence of a typical evaluative priming experiment. The task structure is general. Stimuli (taken from the KDEF, Lundqvist et al., 1998) and masking procedure are examples adapted from Rohr et al. (2012).

When empirical investigations in this field began, researchers focused on the evaluative aspect of stimuli, that is, whether it is positively or negatively connoted, pleasant or unpleasant (e.g., Greenwald et al., 1989; Murphy and Zajonc, 1993; Greenwald et al., 1996), using primarily one specific paradigm: the evaluative priming paradigm. As described above, this paradigm is characterized by the successive presentation of two evaluative stimuli (in the here depicted case: emotional facial expressions; see Figure 1). The target stimulus is task-relevant and needs to be characterized with regard to its valence (i.e., as positive or negative). Preceding the target, a (masked) affective or emotional task-irrelevant stimulus, the prime, is briefly presented (i.e., typically less than 50 ms). It is assumed that automatic processing of the prime stimulus activates an evaluative connotation such that it “primes” the response to the target stimulus, despite its task-irrelevance and masking. Therefore, this paradigm belongs to the broader category of response interference paradigms (Klauer et al., 1997; Wentura, 1999; Wentura and Degner, 2010). The dominant underlying mechanism is that the prime-activated response competes with the response activated by target processing [see also section “Level of Processing and Mechanisms Involved in Masked (Emotion) Priming” below].

The evaluative priming paradigm should not be confused with the semantic priming paradigm (for reviews, see Neely, 1991; McNamara, 2013). In the semantic priming paradigm, prime and target are either semantically related (e.g., butter – bread) or not (e.g., boat – bread). Typically, a lexical-decision or word-naming task is used, requiring the target word to be categorized (as a word vs. non-word) or named (pronounced) as quickly as possible; response latency is typically the dependent variable. In this case, and in contrast to the typical evaluative priming study, the prime variation (i.e., whether the prime is related vs. unrelated to the target) is neutral with regard to the target response (e.g., in the lexical-decision task, both related and unrelated primes are words). Hence, priming effects cannot be explained by response-related processes but must be explained by – generally speaking – encoding facilitation processes. That is, the prime “butter” does not facilitate the “word” response to “bread”, but rather facilitates the encoding and processing of the word “bread” itself (also see below, and see Wentura and Degner, 2010, for a more extensive discussion). Of course, semantic “relatedness” can also be based on evaluative congruence – that is, whether or not prime and target share the same valence. Indeed, there are several studies using this approach (e.g., Spruyt et al., 2012). However, this is a smaller branch of research, and only few studies of this kind have used masked primes. We will return to these studies later.

The history of masked evaluative priming research now spans more than 30 years. Three years after the introduction of the visible evaluative priming paradigm (Fazio et al., 1986), Greenwald et al. (1989) published the first evidence for non-conscious processing of evaluative information.

To date, a considerable amount of evidence for masked evaluative priming has been accumulated, and several features have crystallized as important for masked evaluative priming (for an overview see Table 1). To give the reader an overview, we already shortly mention important factors here. Many of them are outlined in more detail in the following sections (or were already mentioned above). As most critical factors, one might certainly mention the dependent variable, prime duration, (type of) masking and related to these two latter points: stimulus onset asynchrony, that is, the time from prime onset to target onset, because this is the time available for prime processing. Typically, in the masked evaluative priming task, differences in reaction times between incongruent and congruent trials serve as the dependent variable, as mentioned above. The differences in reaction times under masked presentation conditions are typically in the range of approx. 8–25 ms (e.g., Kiefer et al., 2015: RT differences of 7–21 ms; Spruyt et al., 2012, Experiment 1: 13 ms RT difference; Lähteenmäki et al., 2015, Experiments 4–5: 20–30 ms for the affective task), that is, they are numerically small. Effect sizes are also typically small to medium, but heterogeneous effect sizes are reported (e.g., *d*_Z_ = 0.30–1.20; Klauer et al., 2007; see section “Evidence With the Masked Evaluative Priming Task”). Effects can also manifest in differences in errors, depending on specific task parameters, such as response deadline or response window procedures (see Draine and Greenwald, 1998). These instructions demand from participants to answer very quickly and thus push effects into the error rates. Further DVs are, for example, event-related potentials (ERPs) in brain activity or physiological indices, such as, facial muscle responses. Such studies are, however, still the exception in masked evaluative priming.

**TABLE 1:** Important features in masked evaluative priming.

Feature	Know-how	Consequence
Dependent variable	Typically response times (of correct responses) and errors, sometimes dominantly errors (response window procedure; Draine and Greenwald, 1998). Rarely neuropsychological as well as psychophysiological indices (i.e., ERPs and facial muscle activity)	Masked processing is inferred from reaction time/error differences between trials with valence-incongruent prime-target combinations and valence-congruent prime-target combinations.
Prime duration	Typically <50 ms (with backward masking)	Longer durations usually mean more stimulus processing with the risk of reaching visibility
Stimulus onset asynchrony (SOA)	Typically <100 ms (with backward masking)	Effects seem to vanish at longer SOAs (Greenwald et al., 1996), but systematic research in this regard is missing
Type of masking	Typically backward pattern masking by structure or noise	Type of masking determines masking efficiency as well as masking mechanisms, e.g., meta-contrast masking or CFS work differently than backward masking (see sections “The Assessment of Non-consciousness in Masked Evaluative Priming Research” and “Further Research With Masked Affective Stimuli”).
Prime-mask ratio/signal-to-noise ratio	(Typically confounded with SOA)	The longer the mask is presented, the more masking is achieved; however, with the risk of completely suppressing prime processing from a certain point onward; no evaluative priming studies in this regard exist.
Stimulus selection	Words, faces, pictures etc.; rarely auditory stimuli	Most research is done with words or faces; pictures seem to be more difficult to process under non-conscious presentation conditions. Detailed explanations for this empirical finding are still lacking.
(Non)shared prime and target set	Novel or practiced primes	As described in Section “Level of Processing and Mechanisms Involved in Masked (Emotion) Priming,” non-conscious processing with practiced primes relies on different mechanisms compared to processing of novel primes. Providing evidence for non-conscious processing is thus essentially only possible with novel primes.
Stimulus set size/prime and target repetitions		Stimulus set size or the number of stimulus repetitions can have an influence on the underlying mechanisms [see section “Level of Processing and Mechanisms Involved in Masked (Emotion) Priming,”]. Small stimulus sets/many repetitions favor S-R bindings
Awareness measure (see section “The Assessment of Non-consciousness in Masked Evaluative Priming Research”)	Summary or trial-by-trial measure; Non-verifiable self-report or verifiable performance-based measure.	Verifiable performance-based measures typically yield visibility at shorter prime durations than non-verifiable self-report measures. It is still debated which index of (non) awareness best operationalizes (non-)consciousness.

**FIGURE 2:**
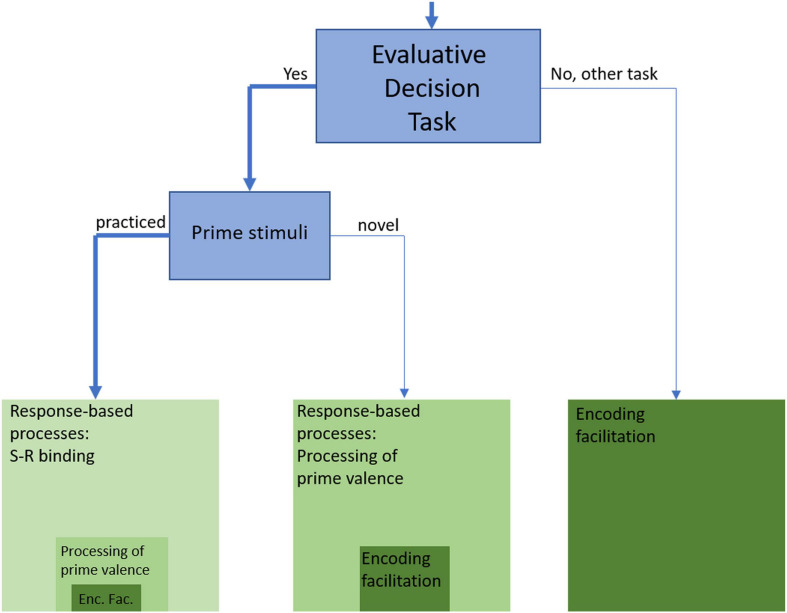
Masked evaluative priming studies and explanations of priming effects (Rectangle size indicates claimed explanatory power; width of the arrow lines indicates the relative number of experiments).

A further important aspect is (type of) masking: the prime is typically masked by a noise mask presented after (i.e., backward masking) or before and after (i.e., sandwich masking) the prime. However, further masking types are possible (i.e., dichoptic masking, continuous flash suppression, and CFS, see below). The specific mask can have a strong influence on processing, which is why researchers differentiate between para-/metacontrast masking, masking by structure, and masking by noise (Agaoglu et al., 2015; see Breitmeyer and Öğmen, 2006, for an overview). For example, Bachmann et al. (2005) have shown that faces are differently masked through quantized (i.e., pixelated) faces or noise masks; even the scale of quantization made a difference. We will not go into the details of this topic, but researchers should be aware that the type of mask does influence masking/visibility, the time course of masking and thus also potential priming effects. In this regard, it is also important to remind of the signal-to-noise ratio or prime-mask-ratio (Agaoglu et al., 2015). Obviously, the longer a mask is presented, the more the masked processing shifts to the processing of the mask and thus noise, with the consequence of weaker prime processing. It is easily imaginable that at some point no signal comes through any more. Typically, this prime-mask ratio in masked evaluative priming is confounded with the stimulus onset asynchrony (SOA), that is, the time between prime onset and target onset and thus the time available for prime processing. In general, evaluative priming is typically only observed for SOAs below 300 ms (Fazio et al., 1986; see also Herring et al., 2013, for the influence of SOA on evaluative priming). For masked priming, the critical SOA period is presumably further reduced. Greenwald et al. (1996) reported that non-conscious priming effects decrease sharply with an increasing SOA beyond 100 ms (Figure 2 in their article actually shows a decrease at an SOA > 67 ms). However, to our knowledge, this is one of the rare masked evaluative priming studies which systematically investigated effects of SOA (see also Andrews et al., 2011; Lähteenmäki et al., 2015; Wentura et al., 2017). Thus, more empirical evidence in this regard might be interesting.

Further factors that should be mentioned are stimulus type, same or different prime and target stimuli (i.e., practiced vs. novel primes), and number of stimulus repetitions. The earlier studies all used word stimuli, while later studies extended the research to facial stimuli (e.g., Banse, 2001; Andrews et al., 2011; Rohr et al., 2012), scene pictures (Hermans et al., 2003) and even sounds (Lähteenmäki et al., 2019). Moreover, it became clear that prime novelty makes a critical difference, as only visibly never-seen stimuli can be claimed to be processed non-consciously in a semantic manner (see below). Likewise, too many stimulus repetitions can prevent non-conscious semantic processing. We outline these points in more detail in Section “Level of Processing and Mechanisms Involved in Masked (Emotion) Priming” and “Evidence With the Masked Evaluative Priming Task.”

### The Assessment of Non-consciousness in Masked Evaluative Priming Research

For evidence of non-conscious processing in the evaluative priming paradigm to be conclusive, the non-conscious status of the prime stimuli must be empirically proven. To this end, participants are often asked (a) either about their subjective perception of the stimuli in the priming task (e.g., Sidis, 1898; Lähteenmäki et al., 2015) or (b) they are instructed to categorize the prime stimulus with regard to an objective feature (e.g., Greenwald et al., 1996). Given this intentional (or instructed) processing of primes, such tasks (objective and subjective ones) are called ‘direct tasks’ compared to the ‘indirect’ priming tasks, in which the primes are not task-relevant and therefore non-intentionally processed.

Phenomenal awareness is typically assessed on the basis of a retrospective, summarized self-report. However, this subjective measurement of consciousness has been criticized for several reasons (e.g., Merikle and Reingold, 1992; Schmidt, 2015): first, the method requires individuals to introspect and (correctly) remember and assess their perceptual impressions. They might, however, be unable to do so or be inaccurate in their estimations. Second, even if individuals are able to access and assess their impressions accurately, they may apply different criteria to infer consciousness, and may therefore require either relatively little or a substantial amount of evidence, depending on the confidence in their impressions (i.e., liberal or conservative responding). Put shortly, self-reports are based on complex introspective processes as well as decision processes which cannot be verified. Therefore, it has been argued that different self-report responses might simply reflect differences in response criteria rather than differences in awareness. Given this problem, some authors have argued that denials of subjective awareness may simply signal weak perceptions of consciousness held with low confidence rather than absence of awareness (Holender, 1986; Macmillan and Creelman, 1991). For example, participants might report subjective unawareness of stimuli but might still be able to discriminate the stimuli in an objective forced-choice task (see below); such a dissociation of perception according to subjective versus objective criteria has been referred to as a “dissociation zone” (Szczepanowski and Pessoa, 2007). As a remedy, objective, performance-based, and verifiable measures of awareness based on signal detection theory have been developed and are sometimes taken as the sole index of (non-)awareness (see, e.g., Merikle and Reingold, 1992). Typically, this involves a task where participants are presented with the same stimulus sequence as in the priming task, but are informed about the primes and are asked to classify them according to a specific feature (e.g., valence, word/non-word, etc.). However, this approach has also drawn criticism: One problem is that the direct test is always administered last (i.e., after the indirect priming task), and participants might be exhausted and/or no longer motivated to concentrate properly on the task. As an alternative, researchers have started to integrate indirect (priming) and direct tests in a single phase, such that participants’ target response is followed by a prime categorization on each trial (see, e.g., Andrews et al., 2011). This solution, however, might also create problems, as the dual-task approach changes the processing characteristics of the priming task (e.g., participants might try to maintain the prime impression in working memory while processing the target).

In sum, there are two general approaches to the assessment of (non)consciousness, involving either subjective (i.e., non-verifiable self-report measures) or objective (i.e., verifiable and performance-based) measures of awareness. These approaches can be further differentiated with regard to specific aspects, such as retrospective or trial-by-trial assessment, and each method comes with its pros and cons. In all measures, non-awareness is inferred based on a specific criterion, for example, subjective reports of non-perception.

In objective, performance-based measures, the absence of awareness is typically inferred from chance performance in the direct test (i.e., *d*′ = 0 for the discrimination of the task-relevant feature; see Schmidt and Vorberg, 2006; Wiens, 2006). If significant priming in the indirect task is nevertheless observed (i.e., an “indirect-without-direct-effect” pattern), non-conscious processing is inferred. This method is, however, not without its pitfalls, because data interpretation rests on specific assumptions about the (objective) direct and indirect tasks and on the difficulty of proving a null hypothesis (Reingold and Merikle, 1988; Greenwald et al., 1995; Klauer et al., 1998a,b; Merikle et al., 2001; Schmidt and Vorberg, 2006; Wiens, 2006). More specifically, clear data interpretation needs to assume that the objective, performance-based measure captures *all* conscious processing (the exhaustiveness criterion) and *only* conscious processing (the exclusiveness criterion; Holender, 1986; Reingold and Merikle, 1988) – otherwise, observed data patterns cannot be interpreted unambiguously. Because these criteria might be difficult or even impossible to satisfy, some authors (Reingold and Merikle, 1988) have put forward the view that it is sufficient to show an “indirect-greater-than-direct-effect” pattern, under the assumption that both tasks are equally sensitive to conscious processing. If the indirect task yields statistically stronger effects than the direct task, then the difference has to result from non-conscious processing. An interesting variant of this approach was proposed by Greenwald et al. (1995), who introduced a regression method: This involves regressing the effects in the priming task on the effects of the direct, objective task; a significant intercept can then be interpreted as evidence for a priming effect in the absence of prime awareness (given an adequate regression procedure, Klauer et al., 1998a,b, and some boundary conditions, see Klauer and Greenwald, 2000). However, there is considerable debate about the regression method (see, Dosher, 1998; Klauer et al., 1998a,b; Merikle and Reingold, 1998; Klauer and Greenwald, 2000; Miller, 2000; Schmidt and Vorberg, 2006). To name an important argument: If no relationship between priming differences and direct prime categorization exists (which is often the case), the intercept simply corresponds to the mean priming difference (because the regression line is parallel to the *x*-axis). Thus, in this case the regression method has no surplus over the report of the tests for priming and direct prime categorization.^1^

To remedy the problem that preconditions are often difficult to prove, Schmidt and Vorberg (2006) suggested to infer non-conscious processing from double dissociations between effects in the direct and indirect tasks. Namely, indirect and direct tasks should differently respond to the same manipulation, such as, for example, a manipulation of the stimulus onset asynchrony (i.e., the time between prime and target onset; see Schmidt and Vorberg, 2006, and see Wentura et al., 2017, for an example with evaluative priming).

Many studies providing evidence for non-conscious processing have used objective measures (e.g., Klauer et al., 2007; Rohr et al., 2012). Thus, one might think that the debate has been settled. However, the (ostensibly) objective measures are still assailable: For example, the standard comparison of direct and indirect effects ignores interindividual variability in awareness, because effects are typically calculated for the whole sample (i.e., the indirect/direct task effect for the whole sample). With this approach, it is thus conceivable that the obtained effects are based mainly on some aware participants. Likewise, it is still possible that this approach includes both trials in which the prime was consciously perceived (that show a priming effect) and trials in which it was not consciously perceived (that do not show a priming effect), so that the calculated effects are based on a mixture of the two. Moreover, the described performance-based measures are often also criticized for ignoring the phenomenal nature of consciousness, which is only accessible subjectively (Wiens, 2006).

This argument, however, returns the spotlight to subjective measures, which have indeed regained importance over the last two decades (see, e.g., Persuh, 2018). In lieu of summarized self-reports, trial-by-trial ratings of subjective impressions have become more common (e.g., Ramsøy and Overgaard, 2004; Szczepanowski and Pessoa, 2007; see Sandberg et al., 2010, for a comparison of several procedures). These can be used to differentiate between aware and unaware trials, and consciousness is typically inferred on an individual trial basis rather than on a sample basis. However, the above-mentioned problem of potential differences in decision criteria remains. Some researchers have addressed this problem by using awareness rating scales rather than dichotomous aware/unaware measures (Ramsøy and Overgaard, 2004). Others have combined the objective (verifiable) and subjective (non-verifiable) measures in each trial to create a subjective signal detection measure (e.g., objectively correct and incorrect responses with high confidence would be classified as hits and false alarms, respectively; Szczepanowski and Pessoa, 2007). Studies using these approaches often find evidence for conscious awareness at shorter prime durations than the earlier studies who assessed participants’ awareness via retrospective self-reports; this has resulted in some being skeptical of whether non-conscious processing of affect exists at all (Lähteenmäki et al., 2015). Problems arise, however, because some researchers regard even just a brief glimpse of a visual impression without conscious access to any content as sufficient for awareness (Ramsøy and Overgaard, 2004; Lähteenmäki et al., 2015), whereas others take the stance that some conscious access to content (i.e., the affective prime) is necessary before one can speak of awareness in evaluative priming (e.g., Zehetleitner and Rausch, 2013).

It is clear that there is no general agreement on what is regarded the most adequate measure and best index of awareness. Consequently, the debate regarding the existence of non-conscious processing of affect is not yet resolved, given that interpretations of evidence depend fundamentally on whether one accepts the employed measures of awareness. Against this backdrop, some discussions have even implied that unsuccessful masking in a masked priming study is the worst of all scenarios: Effects cannot be interpreted as non-conscious, and at the same time power to find effects is decreased by masking. We are reluctant to agree with this viewpoint. Masking – even if imperfect with regard to objective measures of awareness – reduces prime awareness and participant reactibility. Thus, the assumption that effects are caused by automatisms of the cognitive system (as opposed to being strategically produced) can still be upheld, if it can be shown in some way that results differ from the results one would obtain under clearly visible, intentional processing conditions. Such a difference (i.e., the impact of masked implicit processing conditions) is more easily demonstrated and still conveys the most important point, namely that evaluative priming effects arise from automatic processing – even if the exact degree of (un)awareness under which such results emerge can be debated.

Process characteristics that at first glance are not associated with consciousness may provide additional pathways to assess the differences between clearly perceivable primes and masked primes. For example, a phenomenon called the congruence sequence effect is known from structural analogs of response priming such as the Stroop (Stroop, 1935) and flanker tasks (Eriksen and Eriksen, 1974): it means that the congruence effect in trial *n* is moderated by the congruence of trial *n* – 1; for example, the Stroop effect is larger after a congruent trial (e.g., RED written in *red*) than an incongruent trial (e.g., RED written in *blue;* e.g., Kerns et al., 2004). Such congruence sequence effects can be found in evaluative priming as well when using visible primes, but not with masked primes (Frings and Wentura, 2008; also see section “Evidence From the Evaluative Decision Task and Novel Primes,” and Greenwald et al., 1996), which suggests that processes in masked and unmasked conditions are not entirely the same. Thus, it may be more fruitful to focus on such differences in processing rather than debating the best index of awareness, which is likely a futile endeavor given the pros and cons of each method.

In the following, when discussing specific studies, we will nevertheless explicitly mention which awareness measure was used to infer (non)consciousness. We argue that chance performance on a signal detection measure, dissociation of direct and indirect effects, and lack of subjective awareness (i.e., no content awareness of the masked prime) are all plausible indices of non-consciousness.

Before we describe the obtained evidence using such awareness measures, we will elaborate on the processes and mechanisms assumed to underlie evaluative priming effects as not all kinds of apparently evaluative processing should be considered truly processing of *evaluative features.*

### Level of Processing and Mechanisms Involved in Masked (Emotion) Priming

The dominant explanation for evaluative priming effects is to assume that participants tasked to categorize targets as positive or negative also non-intentionally categorize masked primes, which either activates the correct target response (in case of congruence) or the incorrect one (in case of incongruence). Hence, accurate target responses are either facilitated or hampered, respectively, resulting in a priming effect.

Given this backdrop, an evaluative priming effect with masked primes seems to indicate that a stimulus can be non-consciously processed at least to the level of valence (i.e., whether it represents a positive or negative concept). However, more detailed analyses of the masked evaluative priming literature reveal a very important distinction: Many masked evaluative priming studies have used prime and target stimuli that come from the same set (e.g., Greenwald et al., 1996; Draine and Greenwald, 1998; Klinger et al., 2000; Lähteenmäki et al., 2015); that is, the same stimuli were used as both primes and targets, meaning that the stimuli were repeatedly visibly seen and categorized as targets during the priming phase of the experiment.^2^ This may introduce stimulus-response bindings (Damian, 2001) that can be encoded into memory as a consequence of categorizing the visible target stimulus (Logan, 1988; Hommel, 1998). Consequently, Damian (2001) proposed that only stimuli previously presented visibly and mapped onto a response would elicit a masked priming effect. This account is also known as the direct parameter specification account (Neumann, 1990). In a similar vein, Abrams and Greenwald (2000) showed that masked primes can cause effects at the subword-level: in their study, if target words such as *tulip* and *humor* were repeatedly categorized as pleasant, a masked prime such as *tumor* – a combination of the two visibly presented words – subsequently acted as a *pleasant* prime despite representing a clearly unpleasant concept. To the extent that participants had built a stimulus-response binding to the visible target words, the re-combined masked primes can elicit the same response, because participants retrieved the representation of the previously visibly seen words given the overlap in word-fragments. Abrams and Greenwald therefore concluded that non-conscious priming might be rather limited and involve semantic analysis only to a minor degree. That is, according to this analysis, masked primes are *not* processed with regard to their evaluative or affective qualities in these experiments. To demonstrate that affective/evaluative qualities of masked primes are processed, one has to use “novel” primes that are never visibly presented and categorized as positive or negative during the course of the experiment.

However, there are two further qualifications of these rather simple principles. First, the stimulus-response binding does not seem to be located at the level of the concrete motor program, but at the level of the response category: Abrams et al. (2002) trained valence categorization of words in a pre-priming phase, but reversed the target-response key assignments for the priming phase (using the trained words as primes). Nevertheless, congruence effects at the category level were found (e.g., a word whose positive evaluation was associated with right index finger responses in the practice phase subsequently facilitated responses to positive targets made with the left index finger).

Second, Kunde et al. (2003) conceptualized the stimulus-response associations as “action triggers.” This means that participants create a task set when they perform (or are instructed to perform) a specific task, and this task set specifies both the task-relevant stimuli and the associated actions triggered by the stimuli. During the task, only stimuli with an action trigger are processed up to the response level and elicit a response priming effect. Evidence for this account was derived from experiments that are structurally equivalent to the evaluative response priming paradigm: target numbers had to be categorized as below or above five; masked number primes caused a congruence effect, with faster responses if prime and target were both below or above five compared to the incongruent case. Moreover, priming effects were found even if some primes were *not* part of the target set: if participants had to categorize only the specific targets 1 and 4, as well as 6 and 9 throughout the experiment, even the primes 2 and 3, as well as 7 and 8 elicited a priming effect, presumably because these primes lie in the *expected* range of target numbers (i.e., between 1 and 4, or between 6 and 9, respectively); hence, action triggers were created even for these novel primes. In contrast, if participants had to categorize only the specific targets 3 and 4, as well as 6 and 7 throughout the experiment, the novel primes 1 and 2, as well as 8 and 9 did not elicit a priming effect, presumably because these primes were not in the range of target numbers and therefore not in the range of expectation (i.e., participants mentally kept a range of to be expected numbers active ranging from 3 to 7 only).

The original action trigger account by Kunde et al. (2003) cannot be easily applied to the processing of evaluative features of masked primes. Specifically, the account focuses on sharply-defined stimuli from a sparse and limited set. Thus, in numerical priming (see above), even if ‘3’ or ‘three’ is never presented as a visible target, corresponding templates can easily be set up in working memory as a consequence of the task demands (and the associated expected range of stimuli), such that a masked prime matching one of the templates will trigger the associated action. For evaluative priming, such stimulus-specific action trigger templates might be more unlikely given the wide range of affective stimuli that exist; nevertheless, they are possible and should therefore be kept in mind, especially if a small set of novel stimuli is used. If priming effects generalize to broad categories of novel primes, the original action trigger account must make the rather implausible assumption that participants possess pre-existing action triggers for all positive and negative words/images. In a modified account, however, Kiesel et al. (2006) suggested that action triggers can be established at the level of semantic categories. Applied to the processing of evaluative features, this would mean that participants set action triggers for the semantic categories of “good” and “bad.” These action trigger accounts have, however, not been tested with masked processing of evaluative or emotional stimuli (for an exception, see Wentura and Rohr, 2018). Further research is therefore needed to elucidate the mechanisms that are responsible for masked evaluative priming effects under a variety of circumstances.

Figure 2 shows the distinctions we have drawn so far. Any experiment that varies the affective/evaluative congruence of prime and target can be categorized with regard to the task used. In most cases, this is the evaluative decision task, which means we are dealing with response priming tasks that are close neighbors of the Stroop task (Stroop, 1935): a task-relevant feature of the stimulus ensemble –word color in the Stroop task, target valence in the evaluative priming task – is accompanied by a task-irrelevant feature – the color word in the Stroop task, prime valence in the evaluative priming task – that is or is not compatible with the response. If other tasks are used, we are dealing with variants of the semantic priming task, with priming effects that arise due to encoding facilitation processes (broadly defined). We will discuss the semantic priming variants more specifically at the end of the section, but to anticipate, for several reasons they cannot contribute much to our understanding of non-conscious evaluation processes.

Within the class of response priming experiments, the second distinction of importance is whether primes are practiced or novel. Most studies use practiced primes by drawing primes and targets from the same set; by contrast, novel primes are never visibly presented and categorized during the course of the experiment. If primes are practiced, the most straightforward explanation of priming effects is given by the S-R-binding assumption (see above; hence the big rectangle on the left in Figure 1). Of course, it cannot be ruled out that masked primes are non-consciously evaluated (hence the smaller rectangle within the bigger one). Results like those obtained by Abrams and Greenwald (2000; see above) indicate, however, that such evaluation does not play a major role. Thus, the search for non-conscious evaluation processes must be dominantly focused on response priming studies with novel primes (i.e., primes only presented as masked primes).

**FIGURE 3:**
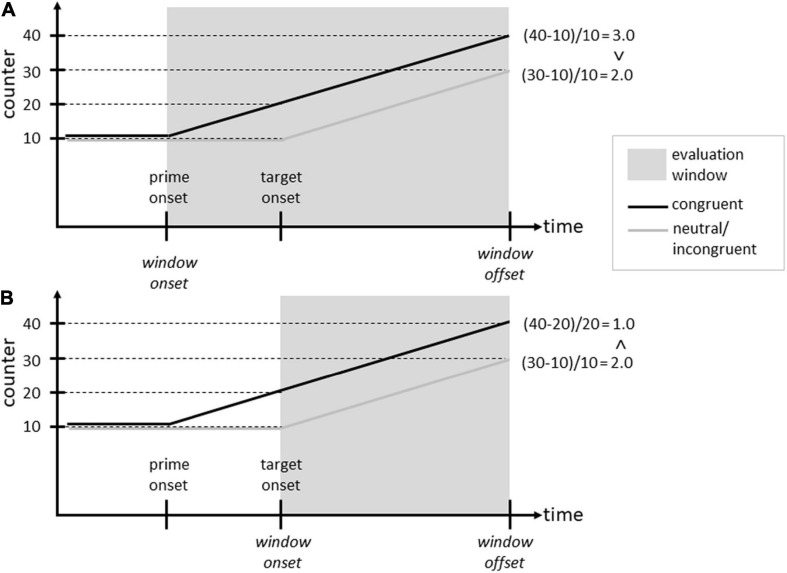
Illustration of the psychophysical account (Klauer et al., 2009). **(A)** The evaluation window includes the prime event; **(B)** the evaluation window excludes the prime event. “Counter” stands for the evidence accumulation in favor of the target response. The arithmetic on the right side exemplifies the counter increases within the evaluation window relativized on the counter at the start of the window. Case **(A)** will result in a positive priming effect; case **(B)** in a reversed effect (see text for further explanation).

We have to add one important complication to the discussion of the response priming experiments (i.e., all experiments using the evaluative decision task, be it with practiced or novel primes): occasionally, reversed effects have been found in (masked) response priming designs (for a review, see Klauer et al., 2009), with *slower* responses in congruent compared to incongruent conditions. These effects can be elegantly explained by a psychophysical account that can be considered a refinement of response-based priming accounts (Klauer et al., 2009; Klauer and Dittrich, 2010). According to this theory, responses in binary decision tasks are based on the *relative increase* of evidence in favor of a response within an evaluation (time) window (see Klauer et al., 2009). Thus, presenting a positive target might increase a “positive” evidence counter, whereas the alternative counter for a negative evaluation might stagnate.^3^ On the basis of the *relative* increase within the window, the positive response will be given. The priming effect will be influenced by whether the evaluation window includes the prime event or starts only after prime offset. In the former case (see Figure 3A), a typical positive priming effect is expected, since the processing of a congruent prime will increase the evidence counter compared to a neutral or incongruent prime, resulting in an overall relative increase that is larger for the congruent condition compared to the neutral/incongruent one. In the latter case (see Figure 3B), however, the prime evidence only increases the baseline at the start of the evaluation window in the case of a congruent prime. Thus, while the absolute increase caused by the target evidence is equal across congruent and neutral/incongruent priming conditions, the relative increase is smaller in the congruent prime condition compared to the neutral/incongruent condition, hence resulting in a reversed priming effect.

What remains to be mentioned is that in the broader evaluative priming literature (which dominantly uses non-masked primes), another general explanatory framework (beyond response-based processes) is discussed with regard to priming effects, based on the mechanisms underlying semantic priming. Notwithstanding the variety of theories in this domain (see McNamara, 2013), the one common core element of interest here is the notion that the prime modifies the processing of the target (and not the preparation of a response; i.e., what we termed above *encoding facilitation*). This general idea is best exemplified by the spreading activation account (Collins and Loftus, 1975; see also Bower, 1991), which assumes that a prime word activates its internal representation, and activation then spreads to related representations. If the target word representation is among these pre-activated representations, it is more accessible than in the control condition. Applied to evaluative priming, this general principle would imply that a positive (negative) prime would render all other positive (negative) concepts more accessible.

A contribution of encoding facilitation to priming effects cannot be ruled out by using the evaluative decision task; however, it is difficult to demonstrate encoding facilitation in this paradigm given the explanatory power of the response-based account. Therefore, as already noted above, encoding facilitation effects can only be unequivocally shown if the task is changed to a lexical decision, naming, or semantic decision task where the target category is orthogonal to valence. There is a long-standing debate on whether encoding facilitation effects can be found at all with such non-evaluative tasks, that is, even with clearly visible primes (see Hermans et al., 1994; Bargh et al., 1996; Giner-Sorolla et al., 1999; Glaser and Banaji, 1999; De Houwer et al., 2001, 2002; Klauer and Musch, 2001; De Houwer and Randell, 2002, 2004; Spruyt et al., 2002, 2004; Wentura and Frings, 2008; Schmitz and Wentura, 2012; Klauer et al., 2016; Werner et al., 2018). While a review of this debate is beyond the scope of this article, for the sake of completeness we will review those (few) studies that have used masked primes in a non-evaluative task.

### Evidence With the Masked Evaluative Priming Task

In light of the reviewed methodological differences relating to the assessment of unawareness and the underlying processing mechanisms, researchers should generally pay attention to whether a given study used primes and targets from different sets, and how it measured awareness. With these two caveats in mind, we will now review the existing evidence in favor of or against non-conscious processing of affect.

#### Evidence From the Evaluative Decision Task and Practiced Primes

As mentioned earlier, evidence for non-conscious processing comes from a considerable number of studies using the evaluative priming paradigm with primes and targets from the same set (or alternatively, studies with primes presented visibly for categorization in a phase preceding the priming phase). Several studies by Greenwald et al. (1989, 1995, 1996), Draine and Greenwald (1998), Greenwald and Draine (1998), and Abrams et al. (2002) provided evidence for non-conscious processing with practiced primes, using either the regression method or an objective measure to establish (un)awareness. Follow-up studies tested thoroughly whether novel primes also elicit non-conscious priming (Abrams and Greenwald, 2000; Abrams, 2005; Abrams and Grinspan, 2007); we will return to these results in Section “Evidence From the Evaluative Decision Task and Novel Primes.”.

The finding of robust masked evaluative priming effects with practiced primes has been replicated by several research teams (Klinger et al., 2000; Klauer et al., 2003; Wentura and Degner, 2010; Neumann and Lozo, 2012; Ye et al., 2014; Kiefer et al., 2015, 2017; Khalid and Ansorge, 2017). These studies fit into a larger body of research using structurally equivalent designs with other categorizations (e.g., numerals; Dehaene et al., 1998; Greenwald et al., 2003; Kunde et al., 2003; gender; Draine and Greenwald, 1998; object size; Pohl et al., 2010). All these studies used the objective, performance-based task (often in combination with the regression method) to establish unawareness of primes. Thus, the study by Lähteenmäki et al. (2015) presents a potential challenge to this work, because Lähteenmäki et al. (2015) questioned the validity of the direct test method. They assessed subjective awareness of primes on a trial-by-trial basis; that is, participants rated subjective prime awareness, using the perceptual awareness scale (Ramsøy and Overgaard, 2004), after each target-related response. The results indicated that priming effects were constrained to trials with at least some prime awareness. However, one potential issue with this study is that it used a comparably long prime duration (i.e., 80 ms), which is typically associated with large detection rates.^4^ As already noted in Section “Level of Processing and Mechanisms Involved in Masked (Emotion) Priming,” all these studies are compatible with the interpretation that S-R-binding caused the priming effects. Thus, these findings do not address the primary question of interest, namely whether non-conscious primes are processed up to the level of valence categories.

We would like to end this section by a reference to a recent study that took a somewhat different – and potentially promising – route to non-conscious processing of evaluative features. Recently, a further priming task – the same-different task – was introduced as a means to study non-conscious semantic processing (see, e.g., Van Opstal, 2021, for a review). In the task, two prime and two target stimuli are presented and participants are required to judge if the two targets are the same or different. If the prime category (i.e., same or different) matches the target category (again: same or different), responses are faster (and more accurate) compared to a mismatch. Up to date, however, only one study targeted emotional valence in this paradigm (Liu et al., 2016). In this experiment, happy and fearful faces were used as primes and targets. Interestingly, in two experiments Liu et al. (2016) found a *reversed* effect, that is, responses were *slower* if the status of the prime pair (i.e., same or different) matches the status of the target pair. Non-consciousness of primes was assessed by an objective direct test that was either conducted subsequently to the priming phase (Experiment 1) or trial-by-trial (Experiment 2). Additionally, a trial-by-trial PAS rating (see section “The Assessment of Non-consciousness in Masked Evaluative Priming Research”) was applied in Experiment 2. How can this new finding be integrated into our review? On the one hand, primes and targets were taken from the same set. Thus, evaluation of each face was practiced after some trials. Therefore, the evaluation itself might be retrieved from memory by the brief presentation of the prime. On the other hand, we cannot apply the S-R binding explanation here because each face serves the role of a target equally often in same and different trials. It is up to further research (e.g., by using novel primes) to fully explore the potential of this new paradigm for assessing non-conscious evaluation processes.

#### Evidence From the Evaluative Decision Task and Novel Primes

As emphasized earlier, the search for non-conscious evaluation processes must be dominantly focused on response priming studies with novel primes (i.e., primes only presented as masked primes). Indeed, there is some evidence that novel primes can elicit masked evaluative priming effects; however, the evidence is less convincing than the evidence from studies using practiced primes.

Early studies (which focused mainly on the effects of practiced primes) reported non-significant results with non-practiced primes (Abrams and Greenwald, 2000; Experiment 3; Abrams and Grinspan, 2007; Experiment 2). However, both those experiments found numerically positive priming effects but had relatively low power (possibly due to the focus on practiced primes, which tend to elicit large effects). For example, in Experiment 3 of Abrams and Greenwald (2000), 9 out of 12 participants showed positive priming (i.e., *p* = 0.07 in a one-tailed significance test). Abrams and Grinspan (2007; Experiment 2) reported a non-significant effect of *d*_Z_ = 0.18 (i.e., a small effect according to Cohen, 1988).

The most rigorous series of studies was conducted by Klauer et al. (2007). In three experiments^5^, Klauer et al. (2007) showed that non-practiced primes caused evaluative priming effects. They did not only use separate prime and target sets, but also accounted for the possibility that priming effects might arise from word segments of the prime retrieving S-R bindings of targets [see section “Level of Processing and Mechanisms Involved in Masked (Emotion) Priming,” the case of *tumor*]. In Experiment 1, the average overlap between primes and targets with regard to single letters, bigrams, and trigrams was larger for incongruent pairings than for congruent pairings; thus, if anything, retrieval of S-R bindings worked *against* the standard congruence effect. In Experiment 2, primes and targets were composed of non-overlapping letter sets. Finally, Experiment 4 used word primes but schematic faces (“smileys” and “grumpys”) as targets. Moreover, performance in the objective direct tests was at chance level. Thus, these experiments showed evidence for genuine masked evaluative processing. The average size of these effects was *d*_Z_ = 0.67 (range *d*_Z_ = 0.30–1.20); thus, effect sizes were medium to large (according to Cohen, 1988), although they were smaller than previously observed masked evaluative priming effects with practiced prime words. Wentura and Degner (2010; Experiment 2) found similar effects with evaluative adjectives and the regression method.

Using adjectives as targets and nouns as primes, Frings and Wentura (2008; Experiment 2) found masked priming effects as well. However, they provided relatively weak evidence for non-consciousness of processes (according to, e.g., Schmidt, 2015)^6^, based on elimination of individual participants with non-random categorization in an objective, direct test (this was 20% of participants; mean *d*′ for the rest was not significantly different from zero). However, most importantly, the authors directly compared masked and unmasked priming with regard to a potential marker of non-consciousness, the above-mentioned congruence sequence effect. Congruence sequence effects only emerged with visible primes, not masked primes, implying non-conscious processing (see section “The Assessment of Non-consciousness in Masked Evaluative Priming Research”).

Kiefer et al. (2015) further specified that priming with novel primes appears to only arise if target stimuli are rarely or never repeated, but not if they are repeated frequently. The authors discussed two theoretical explanations (which are not mutually exclusive): first, targets that are rarely or never repeated (i.e., targets of a large target set) require semantic target analysis (in contrast to frequently repeated targets which might be classified based on retrieval of S-R-bindings), and this level of processing might be needed for masked evaluative effects to emerge (see Kiefer and Martens, 2010, for corresponding semantic priming results). Second, a modified action trigger account [Kiesel et al., 2006; see section “Level of Processing and Mechanisms Involved in Masked (Emotion) Priming”] can explain the effects as a result of executive task-sets at the level of semantic categories if targets do not (or rarely) repeat; in contrast, if targets often repeat, action triggers might be installed at the level of concrete items. Evidence for prime unawareness in Kiefer et al. (2015)’s study was based on objective tests showing *d*’ to not differ from zero.

There are two further studies worth mentioning (Otten and Wentura, 1999; Banse, 2001). Both studies used massively repeated primes, that is, priming conditions involved just one or two different prime stimuli. For example, Banse’s Experiment 2 used the names Charlie Chaplin and Saddam Hussein as positive and negative primes, respectively. Otten and Wentura’s Experiment 2 used the words “figure” and “ground” as primes (which are *a priori* neutral but acquired their evaluative meaning through a pre-priming training phase), in addition to a positive and negative word randomly selected for each participant from a larger set. Both studies showed (replicable) evaluative priming effects. While Otten and Wentura found the typical congruence effect, Banse reported reversed effects (i.e., responses were faster in the incongruent condition). As noted in Section “Level of Processing and Mechanisms Involved in Masked (Emotion) Priming,” reversed effects are occasionally found and can be elegantly explained by a psychophysical account (Klauer et al., 2009). Banse’s experiments had no objective measure of awareness; however, his study can be grouped with other masked experiments in terms of non-consciousness because of the short prime presentation duration (10.5 ms). Otten and Wentura had an objective measure, which showed no deviation of *d*′ from zero (however, the trial number was comparably low in their direct test).

The studies reported so far in this section used words as primes. Experiments with pictures (e.g., scene pictures such as, for example, the IAPS pictures)^7^ have also shown evidence for non-conscious priming, although many of these studies did not control for prime awareness as rigorously as the studies reported earlier. The study by Banse (2001) discussed in the preceding paragraph also had a condition that used the faces (rather than names) of Charlie Chaplin and Saddam Hussein as primes, which likewise yielded a reversed effect, but again no objective measure was employed. Hermans et al. (2003) corroborated these reversed effects with evaluatively polarized pictures (and word targets) in two experiments,^8^ but also did not stringently test for awareness: They used a rather small set of direct test trials and did not force responses and thus could not report *d*′. In a study with school children (*N* = 264; aged 9–14 years), Degner and Wentura (2010; Experiment 2) found masked priming effects (*d*_Z_ = 0.33) with IAPS pictures as primes and targets (prime duration 30 ms; SOA 40 ms), and chance performance in the direct test.^9^ Somewhat critical in the present context, the direct test had only 30 trials. However, given the large sample size, power to detect even a small effect (*d*_Z_ = 0.20) was 1-β = 0.95, with α = 0.05 (one-tailed). Furthermore, Neumann and Lozo (2012; Experiment 1) used disgust-evoking versus fear-evoking IAPS pictures as primes and targets; participants had to categorize target pictures with regard to the two emotions (i.e., a variant of evaluative priming; see also section “Recent Developments: Beyond Good and Bad”). The authors found a congruence effect for novel primes (*d*_Z_ = 0.54), but unfortunately there was no direct test. However, the parameters used (40 ms prime duration, masking with a 50 ms dot-pattern mask) put the experiment within the typical range of masked studies (but see Rohr et al., 2020, who found clear above-chance identification performance with IAPS pictures and comparable parameters, albeit with a different mask).

Several studies have used emotional faces as primes. Andrews et al. (2011) found no evidence for masked priming effects with happy and angry photographic faces.^10^ However, at least in Experiment 1, the authors used a comparably long SOA (293 ms; 80 ms in Experiment 2), and there is evidence that masked priming effects decay rather quickly. For example, Greenwald et al. (1996) found a rapid decline in masked priming effects for SOAs exceeding 100 ms (see also Kiefer and Spitzer, 2000; Kiefer and Brendel, 2006). Other studies have yielded positive evidence for masked emotional face processing (Neumann and Lozo, 2012; Jiang et al., 2013). Jiang et al. (2013) found congruence effects, with chance-level performance in an objective measure test (it is not entirely clear from the report how many trials were used in the direct test). Neumann and Lozo (Experiment 2) corroborated their result of a congruence effect with disgusting versus fear-evoking IAPS pictures (see preceding paragraph) with disgust versus fear faces as primes. Again, they did not employ an objective measure; however, prime duration was relatively short (i.e., 20 ms). In some of our own studies, we used faces from different emotion categories (happy, angry, fearful, and sad) to test for priming effects beyond a simple positive/negative distinction (i.e., participants had to categorize target faces with regard to the specific emotions). While we will return to this specific topic in Section “Recent Developments: Beyond Good and Bad,” here we briefly summarize the main results and whether they can be considered to be based on non-conscious processing. Rohr et al. (2012) found significant congruence effects, which, however, were accompanied by above-chance performance in a direct test. Using the regression approach to prime awareness assessment, the results of Rohr et al. (2012)’s Experiments 2 and 3 (prime duration 14 ms) were, however, found to be compatible with the view that priming occurred under conditions of objective unawareness (see also Wentura and Rohr, 2018). Rohr and Wentura (2014) used spatial frequency-filtered faces (i.e., the high-frequency vs. low-frequency components of face images) as primes. Priming effects were found, with chance-level direct test performance. Using the paradigm of Rohr et al. (2012), Wentura et al. (2017) varied the SOA (43 ms vs. 143 ms, with a prime duration of 14 ms and a mask duration of 29 ms). They observed a double dissociation in the processing of negative emotion aspects (i.e., anger vs. fear/sadness; also see section “Recent Developments: Beyond Good and Bad” below): There was a significant priming effect with a short SOA, accompanied by chance-level performance in the objective direct test; with the long SOA, however, the pattern reversed – priming was completely absent but objective performance was significantly above chance. Double dissociations are seen as particularly strong evidence for process differentiation (e.g., conscious vs. non-conscious; Schmidt and Vorberg, 2006). Interestingly, when priming effects were based on the positive/negative distinction (i.e., happy vs. angry/fearful/sad), they were positively correlated with direct test performance. Overall, it seems that non-conscious processing of face stimuli is a replicable phenomenon, even if the evidence base is not large.

#### Evidence From Variants of Semantic Priming Tasks

As noted above, it has often been speculated that mechanisms of encoding facilitation might also contribute to evaluative priming effects. According to this hypothesis, just as “butter” facilitates the processing of “bread,” any positive (negative) word/picture should facilitate the processing of any other positive (negative) word/picture. A strong test of this hypothesis will require the target response format to be unrelated to the variation of prime-target congruence (e.g., lexical decision or naming task). This question has not yet been answered conclusively for visible primes. Nevertheless, there are some studies that have used masked primes. As for the unmasked versions, evidence is mixed. Klinger et al. (2000), for example, showed that masked evaluative priming effects emerge only when positive/negative responses are required; they found no evaluative priming effects with a lexical decision task (Experiment 2) or an animacy task (Experiment 4). Spruyt et al. (2012) argued that shifting from an evaluative decision to a different task means that the evaluation context may be abandoned, and that this context might be necessary for obtaining encoding facilitation effects (for similar arguments in the context of semantic priming, see Kiefer and Martens, 2010). Therefore, Spruyt et al. (2012) intermixed word-naming trials (25% of all trials) with either evaluative decision trials or semantic categorization trials (i.e., the majority trial type was varied between-participants). Participants were asked to name target words as fast as possible, unless a green rectangle appeared around it, in which case they were required to categorize the target. Interestingly, an evaluative congruence effect in naming latencies was found with the evaluative context but not the semantic context, supporting Spruyt et al. (2012)’s assumptions (for a critical discussion of this paradigm, see Werner and Rothermund, 2013). Evidence for non-awareness was established using the regression method.

To summarize, the past decades of masked evaluative priming research have yielded several insights: First, results can be based on stimulus-response bindings so that the effects do not reflect truly non-conscious processing of evaluative features. Thus, one should pay attention to whether primes and targets are from the same set or not. Second, non-conscious processing of pictorial stimuli (with the exception of faces) produces rather unstable effects, although the evidence base is still rather scarce. Third, and most importantly, non-conscious processing of unpracticed stimuli (words and faces) seems possible, and has been observed even in studies that have applied strict measures of objective awareness as an index of consciousness (Klauer et al., 2007). Thus, in our view, non-conscious processing of affect does occur, but is difficult to prove, as specifics of the experimental procedure need to be taken into account (e.g., SOA; stimulus sets; type of mask; etc.).

#### Evidence From Electrophysiological Studies

Most of the studies on (masked and unmasked) evaluative priming only assessed response times and errors as dependent variables. A few studies, however, additionally reported electrophysiological evidence. We did not integrate these studies into our hitherto existing section taxonomy because EEG studies can potentially be of help to elucidate the role of specific processes [e.g., encoding facilitation, see section “Level of Processing and Mechanisms Involved in Masked (Emotion) Priming” above] even if by design (e.g., response priming) a certain process explanation is favored (i.e., response facilitation or inhibition). To elucidate with a study with visible (i.e., unmasked) primes: Eder et al. (2012) conducted an evaluative decision task experiment with unmasked novel primes and assessed the N400 event-related potential and the lateralized readiness potential (LRP). It is known that semantic processes are specifically reflected in a modulation of the N400 ERP component (Kutas and Hillyard, 1980), a broad negative ERP deflection between 300 and 500 ms over the parietal scalp with larger amplitudes for semantically unrelated words compared with related stimuli in semantic priming studies. The LRP indexes relative response activation in the motor cortex (Coles, 1989). Eder et al. (2012) found evidence for both components, suggesting that both response-related processes as well as encoding facilitation processes might a play a role in response priming versions of evaluative priming (see also Zhang et al., 2006, for further N400 evidence; Bartholow et al., 2009, for further LRP evidence in unmasked evaluative priming).

With regard to *masked* evaluative priming, Kiefer et al. (2017) provided electrophysiological evidence that encoding facilitation processes might play a role in masked evaluative priming using the evaluation task and practiced primes. In two experiments, either picture stimuli (i.e., four positive and four negative IAPS pictures served as primes and targets; Experiment 1) or word stimuli (i.e., four positive and four negative nouns served as primes and targets; Experiment 2) were used in an evaluative decision task with practiced primes. In both experiments, ERPs indexing visuo-motor S-R activation were found. Moreover, for words (i.e., in Experiment 2) additionally a N400 effect was found, in line with Zhang et al. (2006) and Eder et al. (2012). Beyond, Jiang et al. (2018) conducted an evaluative decision experiment with masked primes (emotional faces and novel primes) and made time frequency analyses of the EEG. They found a pattern (i.e., increased midfrontal theta activity and suppressed parieto-occipital alpha activity) that resembles patterns known from other cognitive conflict tasks suggesting as well that response conflict plays a primary role in the paradigm. Unawareness of primes was assessed by an objective, direct test in both studies. Thus, electrophysiological studies help to disentangle the concretely underlying mechanisms further.

## Is It ‘Cold’ Cognitive Processing or Are ‘Hot’ Emotion-Related Processes Involved?

But what about affect or emotion in the non-conscious processing of affective stimuli? The question of whether processing of affective stimuli differs from the processing of affectively “neutral” stimuli has been a prevalent one in the literature on masked affect processing. However, the mechanisms and factors discussed so far are rather general (i.e., types of masking, awareness measures, and specific task parameters). Mechanisms such as semantic processing of masked primes and response conflict are also applicable with non-affective materials. Thus, masked evaluative priming may be seen as simply a “special case” of ordinary semantic category processing. However, there is evidence that indeed something affectively ‘hot’ might be involved in the (non-conscious) processing of affective stimuli. Specifically, the term ‘hot’ refers to affect-related bodily processes such as changes in physiology or brain activity as a consequence of the presented prime stimulus. It is, of course, often a matter of debate whether such reactions are truly related to any specific affect or emotion. Embodiment approaches would argue that such bodily responses – in the context of a presented affective stimulus – are (probably) a partial reinstantiation of a previously experienced emotional response (Niedenthal, 2007). Some emotion researchers, however, might argue that only the synchronized activation of several components makes an emotion an emotion (Moors and Scherer, 2013). An individual bodily indicator should certainly not be over-interpreted; nevertheless, bodily responses to a (visual) affective stimulus can index processes beyond cognitive processing (see also Barrett and Bar, 2009, who argued that affective responses can influence even the early stages of visual processing). In the following paragraph, we will give an overview of the few behavioral studies that have investigated this issue to date (see Tamietto and DeGelder, 2010, for an excellent review of the neural basis of non-conscious processing of emotional stimuli).

Tackling this question is not possible without mentioning Zajonc’s (1980) affective primacy hypothesis and the related research. Specifically, Zajonc proposed that affect and cognition are independent of one another, and that given the importance of affect for survival, affective processing takes priority. He provided evidence for this assumption with a paradigm called ‘affective priming,’ which, however, differs from the masked evaluative paradigm that we have focused on so far. Specifically, Murphy and Zajonc (1993) presented backward-masked affective faces as primes followed by ambiguous Chinese ideographs as targets. Participants’ task was to indicate whether they liked or disliked a presented ideograph on a 5-point scale. Using this paradigm, Murphy and Zajonc obtained evidence for the processing of backward-masked affective faces. Specifically, participants’ likeability responses were biased in the direction of the prime’s valence – suggesting that the affective response to the face was misattributed to the neutral target – although participants could not recognize the faces in a forced-choice awareness measure. Affect-neutral ratings of the ideographs (e.g., ratings of their masculinity/femininity, size, and symmetry) were not affected by the primes. Therefore, the authors concluded that “emotional reactions can occur with minimal stimulation and that they can therefore precede and alter subsequent cognitions” (p. 735). Thus, they took the affect misattribution effect as evidence for ‘hot’ affective processing. However, subsequent research yielded additional insights into the underlying processes (e.g., Murphy et al., 1995; Wong and Root, 2003), revealing that no conscious feelings are induced with this paradigm (Winkielman et al., 1997), and that affective as well as non-affective information can be processed under short and masked presentation conditions (e.g., see Kunde et al., 2003; for non-affective evidence from a response priming paradigm). Moreover, findings of non-conscious processing at very short presentation conditions (i.e., 4 ms, tachistoscopic presentation in Murphy and Zajonc, 1993) could not always be replicated (Kemps et al., 1996; Faivre et al., 2012), raising questions about the reliability of the phenomenon. In addition, several studies have suggested that non-affective semantic information might be processed even earlier than affective information (Storbeck et al., 2006; Nummenmaa et al., 2010), because some semantic recognition of an object would be necessary for affect to be elicited. Consequently, the affective primacy hypothesis was abandoned (also because of some definitional issues; Leventhal and Scherer, 1987). Nevertheless, subsequent research was still interested in the question of whether the early processing of emotional stimuli would involve ‘hot’ affect-related processes.

Indeed, over the years, several studies have provided some evidence for the involvement of “hot” affective processes in both the evaluative priming paradigm and Murphy and Zajonc’s (1993) affective priming paradigm (which in the following we will call the affective misattribution procedure in order to better differentiate it from evaluative priming). For example, using the misattribution paradigm, Rotteveel et al. (2001) showed that facial muscle responses are triggered in response to the masked facial primes – although it should be noted that the faces were presented in a blocked manner (i.e., a number of primes of the same valence were presented consecutively). Results were obtained under conditions of chance performance in a subsequent forced-choice recognition test (with the exception of one participant showing above random responding). Foroni and Semin (2009, 2011) showed that funniness ratings of target cartoons are influenced by masked affective word (2009) or face (2011) primes under conditions of subjective unawareness – but only when participants’ facial muscles were not blocked through a pen-holding manipulation (i.e., participants are instructed to hold the pen with their lips so that the muscles involved in smiling are occupied with this task). Thus, this research suggests that triggered facial responses, as an example of embodied processes (e.g., Niedenthal, 2007), play an important role for such affective priming effects to emerge (also see Niedenthal et al., 2003, for a embodied-cognition argumentation in the context of evaluative priming). Further cues for the involvement of “hot” affect-related processes come from studies involving highly anxious or depressive participants: For example, Li et al. (2008) reported greater misattribution effects in high versus low trait-anxious participants; these effects were accompanied by enhanced electrophysiological differences between positive and negative prime conditions in the P1 component of event-related potentials. Similarly, Gibbons (2009) found a significant response bias in the misattribution task only in high state-anxious participants; in this study, affectively-arousing prime stimuli yielded greater misattribution effects than non-arousing stimuli, thereby also suggesting an influence of ‘hot’ affect-related processes. Further neuropsychological evidence stems from Dannlowski et al. (2007a). They reported that the amygdala reacts to masked affective faces (with blocked presentation) and that this activation correlated with the bias triggered by masked negative face primes in the misattribution task (also see Suslow et al., 2010, for fMRI studies using the masked affective misattribution task). This lab group has also published several studies involving depressive patients, showing some moderation of the misattribution effect by depression (Dannlowski et al., 2006a,b, 2007b; Suslow et al., 2010, 2013).

Of note, in some of these studies, participants were partly aware of the prime stimuli (e.g., Li et al., 2008). Obtaining misattribution effects under conditions of objective unawareness seems thus more difficult (but see Gibbons, 2009; Rohr et al., 2015; for exceptions). Moreover, moderation of evaluative priming or misattribution effects in clinical populations might also stem from cognitive biases in these populations and not from processes related to ‘hot’ affect.

Thus, while there is some evidence for the involvement of affect-related processes in the masked misattribution task, it remains open whether such effects are confined to the masked processing of emotional faces, or whether face processing simply provides the best operationalization to assess non-conscious “hot” affect-related processes (but see Tsikandilakis et al., 2018, for a critical review of the physiological effects of masked emotional face processing). Further research should definitively target other stimulus categories and implement different operationalizations to investigate this issue. It seems likely that “hot” and “cold” processes contribute to affective priming effects to variable degrees, depending on the exact processing conditions, as is the case for the unmasked version of the paradigm (see Payne and Lundberg, 2014, for an overview).

With regard to the evaluative priming paradigm, there is less evidence for the involvement of affect-related bodily processes (under masked presentation conditions). Also, Foroni and Semin (2012) showed that evaluative priming effects only emerged when facial mimicry was not blocked. However, this study used clearly visible presentation conditions. To our knowledge, there are no further studies that tested for an involvement of “hot” affect in the evaluative priming paradigm.

## Recent Developments: Beyond Good and Bad

Recent approaches to the study of non-conscious affective processing have targeted the “hot” versus “cold” issue (alongside some other issues) from a different perspective. In the vast majority of studies, only the valence dichotomy of good (positive and pleasant) versus bad (negative and unpleasant) is assessed. This can be simply seen as a pragmatic constraint. In the evaluative priming paradigm, it is inherent to the task that targets are classified according to valence (i.e., whether they are positive or negative). Thus, by virtue of the task, a simple positive versus negative differentiation of automatic evaluation (here: of the primes) suggests itself. Sometimes, however, it has been postulated that fast automatic evaluations are indeed undifferentiated, that is, restricted to the differentiation of valence (e.g., Zajonc, 1980; Fazio, 1986, 2007; Pratto and John, 1991; Murphy and Zajonc, 1993; Dovidio et al., 2001). Fazio (1986), for example, proposed that (strong) attitudes are associations between objects and summary evaluations; these evaluations are considered merely positive or negative. Others (e.g., Pratto and John, 1991) have argued that screening the environment for dangers and opportunities is fast only at the expense of differentiation.

From a functional perspective, however, this lack of differentiation seems questionable (Wentura et al., 2000; Wentura and Degner, 2010). Specifically, Rohr et al. (2012) as well as Neumann and Lozo (2012) reasoned that, from a functional viewpoint, a pure valence-based differentiation in the early stages of processing makes little sense because stimuli associated with the same valence but different emotions – for example, an enemy showing an angry versus fearful expression – necessitate very different reactions in order to be adaptive (e.g., fight/flight vs. contentment in the example above). Thus, further differentiation of emotion-related appraisals, potentially down to the level of specific emotions, may be pertinent if this early processing serves survival. To examine this issue, Neumann and Lozo (2012) and Rohr et al. (2012) created the emotion priming paradigm, by adapting the evaluative priming task to incorporate the processing of specific emotions. Instead of classifying target stimuli as positive or negative, Rohr et al. (2012) asked participants to classify emotional target faces according to the specific emotion they express (i.e., joy, fear, anger, and sadness). Masked prime faces with emotional expressions (i.e., joy etc.) preceded the target faces. The resulting effects could then be used to differentiate the specificity of processing: If only coarse valence was differentiated, all negative–negative prime-target combinations would be congruent and thus yield shortened reaction times (compared to incongruent combinations), indicating facilitated target processing because of priming. If the specific emotion was processed, then only emotion-congruent prime-target pairs would show this pattern. Rohr et al. (2012), however, found that anger was differentiated from fear and sadness, but the latter two were not differentiated from one another. This pattern of intermediate differentiation is difficult to explain via semantic processing alone. From a semantic processing viewpoint, the priming effect should be valence-based (because the broad categories of positivity or negativity are processed) or emotion-specific (because the specific emotion categories are triggered by the prime). Rohr et al. (2012) thus concluded that early emotion processing is more differentiated than previously assumed (also see Neumann and Lozo, 2012, for an emotion-specific differentiation of disgust and fear), and that specific emotion-related processes seem to be involved, at least to the extent that they facilitate an early estimation of social relevance or coping ability (also see Rohr et al., 2020).

Wentura et al. (2017) showed that this early intermediate differentiation arises only with short SOAs and does not benefit from enhanced prime visibility, suggesting that non-conscious processing indeed underlies this differentiated pattern of results. Of note, however, the differentiated pattern that arises under non-conscious processing conditions depends on the exact processing circumstances: With the same paradigm, but spatial frequency-filtered faces, a more arousal-based differentiation (i.e., sadness differentiated from anger/fear) was observed under conditions of zero awareness (only for low spatial-frequency filtered faces, though; Rohr and Wentura, 2014). Furthermore, with the masked emotion misattribution task (i.e., an adaptation of the masked affect misattribution task), Rohr et al. (2015) also found evidence for this intermediate differentiation. It might be that the stronger contribution of affect-related processes determines the pattern: Low (in contrast to high) spatial frequency-filtered faces are assumed to be closely related to emotion processing (Johnson, 2005; Tamietto and DeGelder, 2010). Likewise, the misattribution paradigm is thought to involve affect-related processes (e.g., De Houwer and Tucker Smith, 2013). To examine this affect-related hypothesis in the misattribution paradigm more directly, we conducted a masked emotion misattribution study in which we assessed facial muscle responses on a trial-by-trial basis in addition to behavioral responses (Rohr et al., 2018). We were able to replicate the results by Rohr et al. (2015), that is, a differentiation of valence and a non-differentiation of anger and fear. More importantly, a multi-level mediation analysis suggested that affect-related processes – indicated by facial muscle responses – play a causal role in the differentiation of valence: Increased zygomaticus major activity, that is, the muscle involved in smiling, and decreased corrugator supercilia, the muscle responsible for brow furrowing, activity following positive primes contributed to the behavioral response choice. Thus, participants seem to rely (partly) on “hot” affect-related processes in the misattribution task. However, these processes only played a minor role for the effect (based on consideration of the beta weights); cognitive, semantic processes were still the predominant factor. It should also be noted that participants were partially aware of the primes in this study. However, under unmasked prime conditions and in the direct test, there was no longer an influence of such bodily processes – under such conditions, individuals seem to rely on cognitive, semantic processes rather than affect-related ones (see also Blaison et al., 2012). From a naïve perspective, this makes perfect sense: if I am focused on processing the external information provided and can clearly see what is going on, I rely on the external input and categorize it. However, if I do not have an intentional external processing focus and external information is vague, I rely more on internal, interoceptive input. However, more research is clearly needed to corroborate this assumption.

An interesting new perspective on non-conscious affective processing is offered by studies on “affective realism” (e.g., Siegel et al., 2018; Wormwood et al., 2019). The basic tenet of this work is that (incidental) affect naturally infuses visual perception. Indeed, using continuous flash suppression (CFS; see section “Further Research With Masked Affective Stimuli” below) as a masking technique, these studies found (masked) misattribution effects similar to those observed by Murphy and Zajonc (1993). Importantly, however, these effects only emerged if the visible target stimulus was presented concurrently with the prime, and not when the affective image preceded the target (i.e., 150 ms SOA). This was taken as evidence for the affective realism hypothesis, but stands in conflict with priming or misattribution explanations. While the affective realism stance makes a strong claim for “hot” affect underlying non-conscious processing, some might call this account “old wine in new bottles.” However, the studies by Siegel et al. (2018) and Wormwood et al. (2019) certainly show that there is still strong interest in this issue, and that further research is needed in order to decipher the “if, when, and how” of the involvement of “hot” affect-related processes.

## Further Research With Masked Affective Stimuli

We have focused on the two most popular behavioral paradigms to investigate the non-conscious processing of affect or emotion: the evaluative priming task and the affect misattribution procedure. By and large, we have thereby also restricted our review to specific processing and masking mechanisms. Several recent misattribution studies, however, have employed a relatively new masking technique: continuous flash suppression, an interocular suppression technique (CFS; Siegel et al., 2018; Wormwood et al., 2019). In continuous flash suppression two different stimuli are presented to both eyes simultaneously: The two be masked stimulus - here: the prime - and the mask, and both compete for awareness. Awareness of the prime is prevented through constant changes in the masking stimulus or stimuli (i.e., every 100 ms) so that attention and awareness remain on this constantly changing information; thereby the name continuous flash suppression. As any technique, CFS comes with its own peculiarities, and the extent of non-conscious processing with this technique is still debated (Pournaghdali and Schwartz, 2020). For example, Hedger et al. (2016) applied CFS to investigate whether threat stimuli gain privileged access to awareness due to their evolutionary importance. However, their results suggest that only fearful faces have such a processing advantage, and that this advantage arises from their low level-features rather than their threat value. Moreover, the accumulating evidence from CFS studies overall tends to speak against any processing outside of awareness (Faivre et al., 2012; Peremen and Lamy, 2014; Hedger et al., 2016; Kleckner et al., 2018). Thus, while CFS represents a new technique to investigate the masked processing of affective stimuli, it has not simplified or solved the issue of whether non-conscious processing of affective stimuli takes place. However, CFS research has highlighted the importance of the specific masking technique used, as the technique may determine how prime processing is interrupted and at what level. Therefore, there might not be one conclusive answer to the questions of whether, when, and how the non-conscious processing of affective stimuli takes place, but several, depending on the masking technique and awareness measure used.

A further recent development in the field of non-consciously processed affective stimuli is the implicit affect primes effort model (IAPE; Gendolla, 2012). A considerable volume of work has provided evidence that masked emotional facial expressions can have an impact on the amount of motivational effort mobilized (e.g., Gendolla and Silvestrini, 2011; Silvestrini and Gendolla, 2011; Freydefont et al., 2012; Chatelain and Gendolla, 2015; Framorando and Gendolla, 2018). Specifically, this work has shown that masked happy and angry facial expressions lead to enhanced effort in cognitively difficult tasks, but relatively less effort in easy tasks (with effort indexed by the associated cardiovascular response), compared to masked sad or fearful facial expressions, which showed the opposite pattern. According to the authors, this pattern of effects is due to the associations of emotion concepts with effortlessness (i.e., happiness and anger) and difficulty (i.e., fear and sadness). Thus, these findings also highlight the possible influence of ‘hot’ affect-related processes on behavioral tasks, from a different angle. In the present context, it is important to note that these effects are constrained to masked presentation conditions; under clearly visible presentation conditions, the effects vanish (e.g., Schüpbach et al., 2014; Framorando and Gendolla, 2018).

## Conclusion, Questions, and Future Developments

Summing up, what has our review revealed? For decades, there has been considerable interest into the masked, presumably non-conscious processing of affective stimuli and its potential consequences. Based on introspection regarding our everyday experiences, the processing of such stimuli can undoubtedly happen outside of awareness, and many studies have revealed that affective stimuli can have an impact on thoughts and behaviors without people subjectively noticing the stimuli, their influence, or the link between them. However, from a scientific perspective – taking into account the debates about the most adequate paradigms, masking techniques, and indices of awareness – the picture that emerges is more differentiated and complicated. In the present review, we focused on the two most popular behavioral priming paradigms (i.e., evaluative priming and the misattribution task), and outlined the parametrical intricacies associated with these paradigms, as well as the associated discussions regarding the (most) adequate indices of non-awareness. In short, our review revealed that there is considerable evidence for the processing of masked affective stimuli; however, if one looks closely, only a minor proportion of studies can claim to have truly implemented non-conscious presentation conditions (i.e., different prime and target sets along with zero objective awareness, a significant intercept with the regression method, or a dissociation of direct and indirect effects). Nevertheless, this handful of studies *has* shown evidence for the non-conscious processing of affective stimuli (e.g., Klauer et al., 2007; Rohr et al., 2015; see section “Evidence With the Masked Evaluative Priming Task”). Moreover, there is also some evidence suggesting that “hot” affect-related, bodily processes can be triggered by masked primes (e.g., Foroni and Semin, 2011; Rohr et al., 2018; see section “Is It ‘Cold’ Cognitive Processing or Are ‘Hot’ Emotion-Related Processes Involved?”).

We know that the debate about whether such non-conscious processing exists and whether it can have “hot” consequences still endures, and some might argue against the conclusions we have drawn, taking different indices or arguments as evidence. Thus, interestingly, the current debate about the existence versus non-existence of non-conscious processing may best be described as one of definitions rather than a debate about the results of existing studies (e.g., a debate about acceptable measures of awareness rather than a debate about empirical results obtained with the various indices). Thus, considering the presented evidence, we think that one can clearly advocate in favor of the existence of non-conscious emotion processing. Someone who is skeptical of the employed awareness measures may remain unconvinced, but will find themselves confronted with method-related problems that might prove impossible to overcome entirely (e.g., the exhaustiveness and exclusiveness problems). Certainly, researchers should pursue dual avenues: they should (a) aim to improve (methodological) criteria for (non)consciousness, such as finding a clear dissociation between conscious and non-conscious processing instead of merely providing evidence for (non)awareness, and (b) aim to empirically improve our understanding of non-conscious emotion processing. We hope that the present review can contribute to these pursuits.

Thus, to set the stage for future research: If, for a moment, we take non-conscious processing of affective stimuli for granted, where should the field move in the coming decades? Firstly, our review made clear how important tiny technical parameters can be. Research will need to put more emphasis on these small but crucial factors to avoid drawing unwarranted conclusions, and also to allow researchers who are less familiar with the field to (a) implement masked research employing adequate parameters and (b) more easily decide whether (non-)significant results simply reflect inappropriate experimental parameter settings (for further discussion of the importance of technical details in masked priming, see Rohr and Wagner, 2020).

Secondly, our review has highlighted that the assessment of whether or not non-conscious processing takes place critically depends on the awareness measures that one accepts as an index of (non-) awareness. Typically, a greater range of processing is possible under subjective unawareness compared to objective unawareness. For the various objective awareness measures, however, it is difficult if not impossible to decide whether exclusiveness and exhaustiveness are given. That is why Schmidt (2015; see also Schmidt and Vorberg, 2006) makes a strong argument for finding dissociation effects. We argue that the field should accept several indices of awareness, but elucidate more on which mechanism or level of the processing observed effects might be based. Therefore, one might rely on a taxonomy of processing, such as the one proposed by Dehaene et al. (2006), and relate the employed masking technique and awareness measure to the taxonomy (e.g., summarized self-report indicates probably some sort of preconscious rather than non-conscious processing). Researchers who are experts in masking and the underlying mechanisms might even provide a more encompassing taxonomy that spans all currently available masking measures.

Questions worthy of further exploration relate to (a) the specificity of masked processing of emotional stimuli, (b) the issue of whether and when ‘hot’ affect-related bodily processes are involved in the masked processing of affective stimuli, and (c) what kind of stimuli can be processed non-consciously (e.g., pictures). Concerning the first issue, it is still not clear under what conditions an intermediate differentiation will arise and when the differentiation will be along valence and social relevance, or valence and arousal dimensions (Rohr et al., 2015, 2020). It is possible that affect-related versus semantic processes might differentially contribute to the pattern of effects observed. However, the focus of processing (i.e., whether or not it depends on a social processing mode; Wirth and Wentura, 2019) or other influences might also be responsible for the emergence of specific effect patterns. Indeed, if one contrasts only two specific emotions in a priming paradigm, processing of the specific emotions seems possible under masked processing conditions (with marginal awareness; Wentura and Rohr, 2018). Further research is definitively needed to more conclusively address questions regarding the specificity of masked processing of emotional stimuli.

With regard to the involvement of ‘hot’ affect, more research is needed to elucidate the conditions under which bodily, affect-related responses are triggered and contribute to evaluative or emotion priming effects. Social relevance or task relevance seem to be important factors (e.g., Weinreich and Funcke, 2014), and so are automatic (masked) processing conditions, as opposed to unmasked, more strategic presentation conditions (e.g., Rohr et al., 2015). One reason why research into this issue has been sparse may be that it is difficult to demonstrate the contribution of affect processes in contrast to the predominant cognitive and semantic processes (see Rohr et al., 2018).

With regard to the question of what stimuli can be processed outside of conscious awareness, one notion we have not discussed so far is that the stimuli that produce the clearest evidence of subconscious processing are stimuli for which people generally have great expertise (Bruce and Young, 2012) and for which clear mnemonic representations exist, namely faces and words. It may perhaps therefore be the case that stimuli can only be processed non-consciously up to a semantic level if a mental representation exists in memory that can be activated based on such transient input. If, however, an individual is confronted with an object or scene they have never seen before, the masked input might be insufficient to have any impact.

Naturally, this list of open issues is incomplete and biased by the present review’s focus on behavioral paradigms. Thus, we hope that future research will generate new questions and elucidate further the ‘if, what, and how’ of the non-conscious processing of emotion. We hope that our review will inspire new research into this topic, and has made it clearer that the non-conscious processing of emotion is a reliable phenomenon despite the pitfalls and methodological intricacies that come with its empirical exploration.

## Author Contributions

MR conceived the review. MR and DW wrote substantial parts of the manuscript. Both authors contributed to the article and approved the submitted version.

## Conflict of Interest

The authors declare that the research was conducted in the absence of any commercial or financial relationships that could be construed as a potential conflict of interest.

## Footnotes

1 Of course, the test whether the intercept is greater than zero becomes weaker (compared to the test of the mean priming difference itself) the more the mean of the direct test is above zero, since the confidence interval of the regression increases with the deviation from the mean. One might see this as a kind of “penalty” for any deviance of the direct test from zero. However, beyond an intuitive plausibility, this idea lacks a clear rationale.

2 Of course, there is typically a constraint to ensure that prime and target are not identical on a given trial.

3 … or decrease, if we assume that in a binary decision situation, the two counters may be negatively correlated. However, this detail is not important here.

4 The authors additionally used a prime duration of 10 ms (which is relatively low). At this duration neither priming nor awareness were observed.

5 A fourth experiment used a gender-classification task and corresponding materials.

6 Such a proceeding is relatively weak because it disregards that the prime was visible for some participants so that results might be based on a sampling fallacy.

7 The International Affective Picture System: a collection of positive and negative pictures (Lang et al., 2008).

8 Their first experiment with pictures as primes and targets did not show an effect.

9 Note the study was mainly focused on automatic prejudice effects in children. Therefore, the conditions of primary interest used faces of young German and Turkish men as primes. However, IAPS pictures served as a reference condition to show that the typical evaluative priming effect can be found in children.

10 Experiment 1 additionally used schematic faces, however, with only a rather small number of trials.
