# Corrigendum: Degree and Complexity of Non-conscious Emotional Information Processing – A Review of Masked Priming Studies

**DOI:** 10.3389/fnhum.2021.751707

**Published:** 2021-09-01

**Authors:** Michaela Rohr, Dirk Wentura

**Affiliations:** Department of Cognitive Psychology, Saarland University, Saarbrücken, Germany

**Keywords:** (non-)consciousness, emotion, affect, masked priming, evaluative priming

In the original article, there was an error. We wrongly stated that participants in the study of Gibbons (2009) were partly aware of the prime stimuli. This was incorrect. In this study, objective unawareness was given.

A correction has been made to the section: Is it ‘cold’ cognitive processing or are ‘hot’ emotion-related processes involved?, Paragraph 4. The corrected paragraph is below:

Of note, in some of these studies, participants were partly aware of the prime stimuli (e.g., Li et al., 2008). Obtaining misattribution effects under conditions of objective unawareness seems thus more difficult (but see Gibbons, 2009; Rohr et al., 2015; for exceptions). Moreover, moderation of evaluative priming or misattribution effects in clinical populations might also stem from cognitive biases in these populations and not from processes related to ‘hot’ affect.

The authors apologize for this error and state that this does not change the scientific conclusions of the article in any way. The original article has been updated.

## Publisher's Note

All claims expressed in this article are solely those of the authors and do not necessarily represent those of their affiliated organizations, or those of the publisher, the editors and the reviewers. Any product that may be evaluated in this article, or claim that may be made by its manufacturer, is not guaranteed or endorsed by the publisher.
